# Modification of Adenovirus vaccine vector-induced immune responses by expression of a signalling molecule

**DOI:** 10.1038/s41598-020-61730-8

**Published:** 2020-03-31

**Authors:** Christine S. Rollier, Alexandra J. Spencer, Karen Colbjorn Sogaard, Jared Honeycutt, Julie Furze, Migena Bregu, Sarah C. Gilbert, David Wyllie, Adrian V. S. Hill

**Affiliations:** 1 The Jenner Institute, University of Oxford, Old Road Campus Research Building, Roosevelt Drive, Oxford, OX3 7DQ UK; 2 Present Address: Oxford Vaccine Group, University of Oxford, CCVTM, Churchill Hospital, Oxford, OX3 7LJ UK

**Keywords:** Lymphocyte activation, Malaria

## Abstract

Adenoviral vectors are being developed as vaccines against infectious agents and tumour-associated antigens, because of their ability to induce cellular immunity. However, the protection afforded in animal models has not easily translated into primates and clinical trials, underlying the need for improving adenoviral vaccines-induced immunogenicity. A Toll-like receptor signalling molecule, TRAM, was assessed for its ability to modify the immune responses induced by an adenovirus-based vaccine. Different adenovirus vectors either expressing TRAM alone or co-expressing TRAM along with a model antigen were constructed. The modification of T-cell and antibody responses induced by TRAM was assessed *in vivo* in mice and in primates. Co-expression of TRAM and an antigen from adenoviruses increased the transgene-specific CD8+ T cell responses in mice. Similar effects were seen when a TRAM expressing virus was co-administered with the antigen-expressing adenovirus. However, in primate studies, co-administration of a TRAM expressing adenovirus with a vaccine expressing the ME-TRAP malaria antigen had no significant effect on the immune responses. While these results support the idea that modification of innate immune signalling by genetic vectors modifies immunogenicity, they also emphasise the difficulty in generalising results from rodents into primates, where the regulatory pathway may be different to that in mice.

## Introduction

The developing world urgently needs new vaccines for diseases such as malaria, HIV-AIDS and tuberculosis, which together kill over 5 million people every year^1^. Non-replicating recombinant viral vectors have been developed as promising new vaccine candidates. Adenovirus based vaccine vectors induce both innate and adaptive immune responses in mammalian hosts^2–4^. They continue to be developed because of their well-recognised ability to induce potent cellular as well as humoral responses against transgenic antigens from target pathogens. However, the protection afforded by such vaccines in animal models has not translated readily into primates^5^. Therefore, attempts are being made to increase their immunogenicity and efficacy by combining them with other vaccine formulations and heterologous booster doses, or by engineering the inserted transgenes.

The understanding of antigen presenting cells (APCs) suggests that early interactions with T lymphocytes have profound effects on immune activation^6^. The activation state of the APCs is a key factor in the activation of T-cell responses and is influenced by environmental signals, which include the sensing of pathogens through pattern-recognition receptors (PRRs)^7^. The downstream patterns of signalling from PRRs are increasingly understood, with four key intracellular activating adapters identified which are transducers of Toll like receptor signalling. These adapters couple receptors to the induction of proinflammatory cytokine signalling, and in some cases interferon signalling^7^. One of these, the TRIF-related adaptor molecule (TRAM), activates both interferon and proinflammatory pathways linked to Toll-like receptor (TLRs) 4 and 2, in response to stimuli including bacterial lipoproteins, LPS, and large DNA viruses^8,9^.

We have shown *in vitro* in mammalian cells that expression of TRAM from plasmids in transient transfection assays activates the *cxcl2, lcn and ifnb (*interferon b) promoters, which are canonical early and late pro-inflammatory pathway and interferon signalling pathways target genes, respectively^10^. When co-expressed with the TAK1 (MAP3K7) serine/threonine kinase, TRAM expression increases T-cell responses induced by DNA vaccination *in vivo* in mouse^11^. We hypothesized that the expression of TRAM from an adenovirus vector could alter the virus-dependant signalling cascade *in vivo*, modifying viral immunogenicity. We investigated immune responses elicited by adenovirus vaccine immunization, with and without TRAM expression, in both murine and non-human primate models.

## Materials and Methods

### Plasmids, recombinant adenoviruses and recombinant MVA vaccines

**Figure 1:**
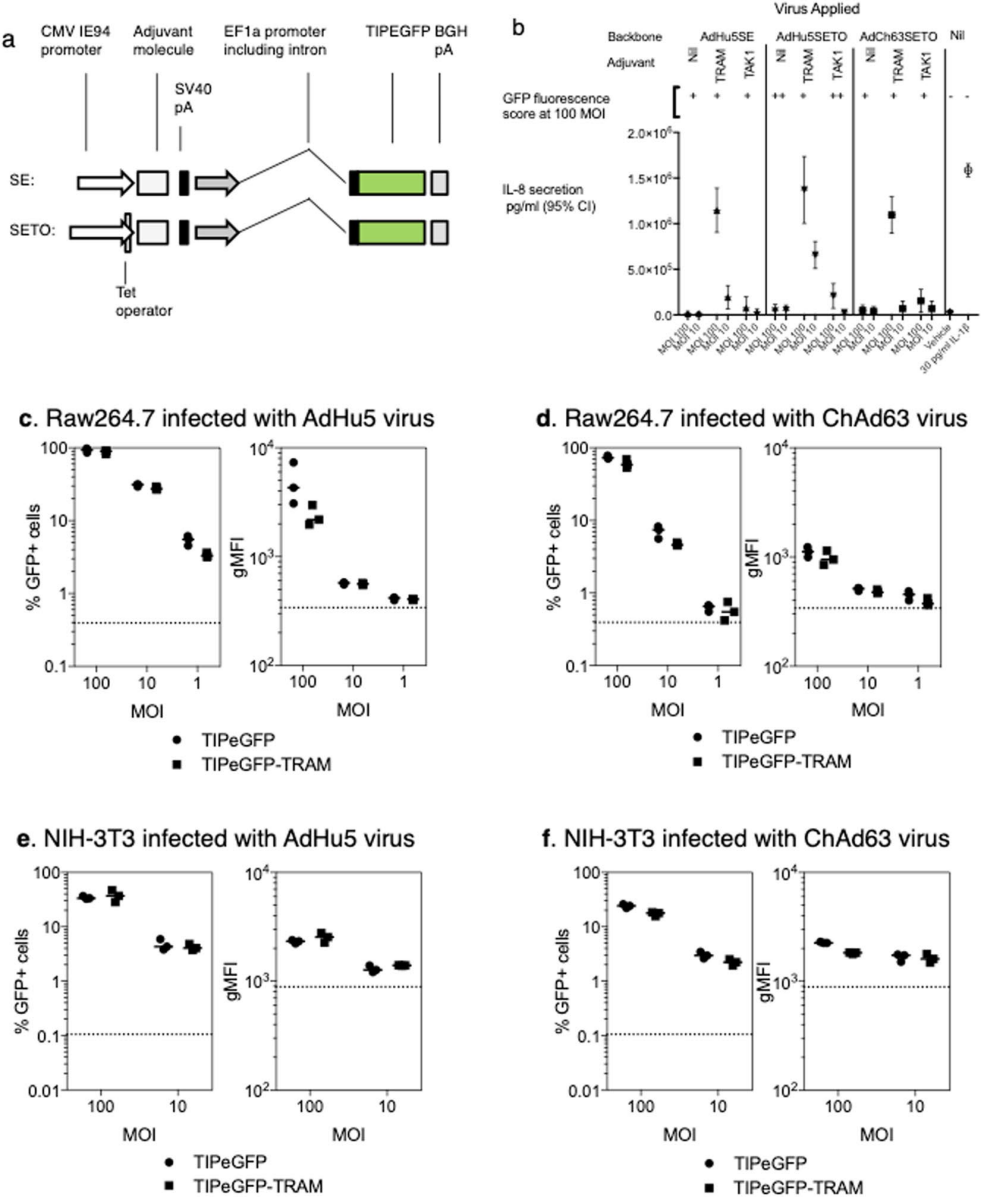
TRAM-induced inflammatory signaling and adenovirus expression cassettes. (**a**) AdHu5 and ChAd63 adenoviruses were constructed by incorporating expression cassettes (illustrated) into the deleted E1 region. Two cassettes were studied, SE and SETO, the latter featuring repression of CMV promoter function during viral growth. All viruses expressed a model antigen (TIPEGFP) driven by the human EF1α promoter**. (b**) TRAM expression induces IL-8 response *in vitro* HeLa cells were either exposed to adenoviruses at an MOI of 10 or 100, or stimulated with IL1β, or exposed to vehicle (Optimem medium) for one hour. 36 hours after infection, antigen expression was assessed by microscopy and IL-8 secretion measured by EIA. **(c–f**) Co-expression of mouse TRAM does not increase the antigen transgene expression in mouse cell lines. Raw246.7 (**c,d**) or NIH-3t3 (**e,f**) cells were infected with the AdHu5 (**a,e**) or ChAd63 (**d,f**) at an MOI of 100, 10 or 1. Cells were harvested 24 hours later and the level of GFP expression measured by flow cytometry. Graphs represent the percentage of infected cells (GFP+) or the geometric mean fluorescence intensity (gMFI) of the GFP signal, each dot represents a replicate well with lines representing the median response.

The synthetic multiepitope string (TIP, Tuberculosis-Immunodeficiency virus-*Plasmodium* epitope), containing CD8+ and CD4+ T cell epitopes from *Plasmodium berghei*, *Mycobacterium tuberculosis* and SIV, was described previously^11^. To generate recombinant adenoviruses expressing both the TIP antigen and an immune activator, a Gateway-compatible bicistronic entry vector was generated, derived from pENTR4 (Fisher) and the bicistronic expression vector previously described^11^ by a series of conventional cloning steps. To permit monitoring of antigen expression, the TIP antigen with a TIP antigen was fused in-frame to the N-terminus of GFP by PCR amplification and restriction cloning. One of the CMV IE94 promoters was replaced with the powerful human elongation factor 1α promoter (phEF1) from pEF-BOS^12^ to minimise repetitive sequences in the vector. phEF1 was used to drive antigen expression, while the CMV IE94 promoter was used to drive expression of mTRAM, and other inflammatory activator ORFs (Fig. 1a). In the experiments described here, two almost identical designs of this bicistronic expression cassette were used. One, designated the SE cassette, is described above. The SETO cassette differs from the SE cassette by addition of a Tetracycline-operator containing sequence flanking the CMV promoter TATA box (Fig. 1a) to limit CMV-driven expression in Tetracycline repressor containing cells^13^.

SE and SETO expression cassettes were transferred from their plasmids of origin into the E1 locus of pAd/PL-DEST (Invitrogen), or the ChAd63 (chimpanzee adenovirus type 63) vector^14^ by *in vitro* recombination using LR clonase (Invitrogen). Control ChAd63 adenoviruses expressing blood-stage malaria antigens Pfm128 and PfAMA-1 or an irrelevant antigen (secreted alkaline phosphatase SEAP) were also used (hAd5-Pfm128, ChAd63-PfAMA-1 and ChAd63-SEAP). A monocistronic chimpanzee adenovirus serotypes serotype 63 (ChAd63) vector in which the complete human TRAM open reading frame was expressed by a CMV promoter was also created (ChAd63-TRAM), as previously described^15^. All adenoviruses were grown in human embryonic kidney 293 (HEK293) cells, purified by CsCl gradient ultracentrifugation, and dialyzed against 10 mM Tris (pH 7.4). Plasmid sequences were confirmed by Sanger sequencing, and are included in Supp. Data 1. In studies of both mouse and human TRAM, the entire unmodified open reading frame was expressed, including the myristoylation signal. The viruses were titered as described previously^13^ on HEK293 cells grown under standard conditions. Wells were scored positive or negative 10 days after infection using hexon immunostaining (Cell Biolabs Inc, San Diego, CA, USA), and infectious unit titers per milliliter were calculated with the Spearman-Karber method. A recombinant modified vaccinia virus Ankara (MVA) vector encoding ME-TRAP was also used, and was described previously^16^.

### Viral bioactivity and antigen expression

To assess bioactivity of the viruses generated, HeLa cells were grown in DMEM containing 10% heat inactivated fetal bovine serum in 96 well plates to 80% confluency (~1 × 10^5^ cells per well). Three treatments were compared: no stimulus, infection with various recombinant viruses, application of IL-1β. In all cases, media was aspirated and 100μl Optimem medium (Gibco) containing the stimulus was applied for one hour. For viral infection, medium contained virus at 10 and 100 MOI. For cytokine stimulation, media contained 30 pg/ml IL-1β (Calbiochem). GFP production was scored visually using an inverted fluorescence microscope 35 hours post infection. Thirty six hours post infection, supernatants were aspirated and IL-8 quantified in the supernatants (DY208, RnDSystem DuoSet).

To assess transgene expression quantitatively, Raw246.7 or NIH-3t3 cells, grown in DMEM containing 10% heat inactivated fetal bovine serum, were seeded (5 × 10^4^ Raw246.7 and 2 × 10^4^ NIH-3T3 cells per well) into 96-well flat bottom plates and left to adhere overnight. The following day, cells were infected with AdHu5-TIPEGFP (human adenovirus type 5, expressing fused to EGFP: enhanced green fluorescent protein) and ChAd63-TIPEGFP vectors, with and without TRAM, at increasing MOIs. 24 hours later, culture media was removed from each well and 30 µL of trypsin (TrypLE Express Enzyme, Life Technologies) added and incubated for 10–15 minutes, prior to resuspension of cells in 10% FCS in PBS and acquisition.

### Immunizations

Mice were used in accordance with the UK Animals (Scientific Procedures) Act under project license number 30/2414 granted by the UK Home Office. The experimental protocols were approved by the Oxford University institutional Animal Welfare and Ethics Review Committee (AWERB), prior to getting licensing from the UK home Office. For induction of short-term anaesthesia, animals were either injected intraperitoneally (i.p.) with ketamine and Domitor® or anaesthetised using vaporised IsoFlo®. Female BALB/cOldHsd (BALB/c) or C57BL/6OldHsd (C57BL/6) mice (Harlan UK Limited) were immunised intradermally (i.d., into both ears) or intramuscularly (i.m., into each of the tibialis muscles of the hind limbs) with 50μl of vaccine in endotoxin-free phosphate-buffered saline (PBS). Groups of a minimum of four mice were used. The number was based around power calculations in which the variable under study (such as IFN-gamma secreting cell numbers) was log normally distributed with standard deviation of 10% of the mean, as based on previous data, and a 80% power to detect a 2.5 fold difference.

Male rhesus macaques (Macaca mulatta) aged between 4 ½ to 8 ½ years were housed at the National Primate Research Centre and cared for according to the regulations and guidelines of the University of Wisconsin Institutional Animal Care and Use Committee. The experimental protocol was reviewed and approved by the Wisconsin Institutional Animal Care and Use Committee (IACUC). Animals were anesthetized for all procedures and were returned after completion of the study into the colony for potential use in other studies or breeding programs. Male rhesus macaques were initially screened for neutralising antibodies against ChAd63 and background ELISpot responses to TRAP and ME peptides. Selection of 6 animals per group was based on ensuring an equal weight and age distribution as follows: negative control group 1 (ChAd63-ME TRAP) 8.8 kg (range 7.3–11.7 kg) and 5.9 years (range 5.3–7.3); group 2 (ChAd63-ME TRAP and 5x ChAd63-hTRAM) 8.5 kg (range 6.9–9.8) and 5.8 years (range 5.0–7.0); control group 3 (ChAd63-ME TRAP and 5x ChAd63-PfAMA-1) 8.2 kg (range 6.7–10.2) and 5.9 years (range 5.3–6.9). Animals received 1.5 × 10^8^ iu of ChAd63-ME TRAP (8.9 × 10^9^ vp) alone or mixed with 7.5 × 10^8^ iu ChAd63-hTRAM (4.1 × 10^10^ vp) or 7.5 × 10^8^ iu ChAd63-PfAMA-1 (7.2 × 10^10^ vp), in a total of 0.3 ml into the deltoid muscle at weeks 0 and 8. Blood samples were taken on the day of vaccination and at weeks 2, 4, 8, 10, 12, 16 after the first ChAd63 vaccination. All animals received 5 × 10^7^ pfu MVA.ME TRAP at week 16 and blood samples were collected at weeks 17 (1 week post MVA), 19 and 23.

### *Ex vivo* IFNγ ELISPOT

Mouse blood or mashed and filtered spleens were used for enumeration of antigen-specific IFN**γ**-producing T cells as described^11^. Nonhuman primate specific IFN-γ ELISPOT assays were performed as previously described^17^ using precoated ELISpot^PLUS^ kits according to the manufacturer’s recommendation (Mabtech USA, Mariemont, OH, USA). All tests were performed in duplicate using pools of 20-mers at a final concentration of 5 μg/ml. Background values (mean of spots without peptide) were subtracted. A response was considered positive if the mean number of SFCs (spot forming cells) of duplicate sample wells exceeded background plus two standard deviations (STD) and was >50 SFC per 1 × 10^6^ cells.

### *Ex vivo* memory and intracellular cytokine staining

Frozen PBMC samples were thawed and rested for 6 hours at 37 °C in media containing 10U/ml of Benzonase (Novagen). Cells were then stimulated for a total of 16 hours with a single pool of TRAP peptides at 2 μg/ml final concentration, anti-CD28 APC (clone CD28.2, eBioscience), anti-CD49d (clone 9F10, eBioscience), anti-CD107a PECy5 (clone eBioH4.A3, eBioscience), Golgi-Stop and Golgi-Plug (BD Bioscience). Surface staining with CD95-bi (clone DX2, eBioscience) av-qDot565 (Invitrogen), CD4-APC H7 (clone L200, BD Bioscience), CD14 PE-Cy7 (clone M5E2, BioLegend), CD20 PE-Cy7 (clone 2H7, BioLegend) and live-dead Aqua was followed by fixation in 10% neutral buffered formalin (Sigma), prior to intracellular staining for CD3-PE (clone SP34–2, BD Bioscience), CD8 APC-H7 (clone RPA-T8, eBioscience) and IFN-γ FITC (clone 4 S.B3, BioLegend) in Perm-Wash Buffer (BD). All flow cytometry data were analysed using FlowJo (TreeStar) with cells gated based on size, doublet negative, CD3^+^ CD14/CD20^−^, and either CD4^+^CD8^−^ or CD4^−^CD8^+^.

### Evaluation of antibody responses

Antibodies to GFP were measured by ELISA. Briefly, 96 well Nunc-Immuno Maxisorp plates were coated for 1 hour at room temperature with recombinant GFP (Millipore) at a concentration of 2 μg/ml diluted in bi-carbonate buffer (Sigma Aldrich). After blocking with 1% BSA in 0.5% Tween-20 PBS, serum was incubated for 2 hours prior to detection of bound antibodies with alkaline phosphatase-conjugated goat anti-mouse IgG (whole molecule) (Sigma Aldrich) diluted 1:5000 and development with NPP substrate (Sigma Aldrich). Serum antibody endpoint titres were taken as the x-axis intercept of the dilution curve at an absorbance value 2x standard deviations greater than the OD_405_ for naïve mouse serum (typical cut-off OD_405_ for positive sera = 0.15). Serum from naïve mice were pooled and used as controls for all the ELISAs and were always below the level of detection.

Antibody responses to TRAP were measured using a luciferase immunoprecipitation system (LIPS) as previously described^18^. Briefly, serum samples were incubated for 1 hour with a cell lysate from 293 cells transfected with a TRAP-rLuc expression plasmid, prior to incubation with Protein A/G UltraLink Resin beads (ThermoScientific) in MultiScreen HTS membrane Barex plates (Millipore) for 1 hour. Unbound lysate and antibodies were removed by washing the plates prior to quantification of bound rLuc activity using *Renilla* luciferase assay system (Promega) and a Varioskan Flash luminometer (Thermo). Antibody levels are expressed as a fold increase from the pre-vaccination timepoint.

### Statistical analysis

GraphPad Prism version 5.0 was used to perform two-way ANOVA testing when multiple doses or routes were tested.

## Results

### TRAM expression from a bi-cistronic adenovirus vector increases IL-8 production *in vitro*

Bi-cistronic AdHu5 vectors and ChAd63 vectors encoding a model transgene of interest (containing malaria and TB epitopes, fused to EGFP), driven by a human EF1α (hEF1α) promoter, were constructed including cassettes allowing inflammatory signalling components to be expressed under the control of a CMV promoter (Fig. 1a), while the antigen was driven by an hEF1α promoter, a strong mammalian promoter^12^.

**Figure 2:**
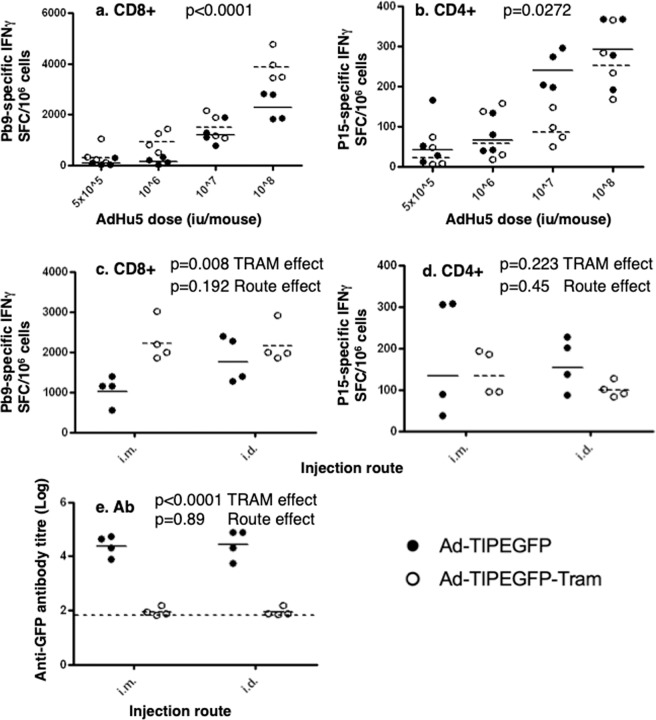
Co-expression of TRAM in AdHu5-TIPEGFP vector impacts on the transgene-specific immune responses. Groups of BALB/c mice were immunized i.m. at day 0 with AdHu5-TIPEGFP (SE) (black dots, black lines in all graphs) or with AdHu5-TIPEGFP-tram (SE) vectors (open circles, dotted lines in all graphs), at the doses indicated **(a,b)**, or at 4 × 10^8^ iu/mouse **(c–e)**. Specific CD8^+^ T cell responses to Pb9 peptide (**a,c**) or CD4+ responses to P15 peptide (**b,d**) were measured by IFN-γ ELIspot at week 2. Antibody titres against GFP protein were measured at the same time point (**e**) and the long dotted lines represent the detection limit of the assay. Each dot represents one mouse and the short lines represent the geometric means per group. P values are indicated (2-way ANOVA).

Infection of HeLa cells by these vectors showed that TRAM-expressing vectors consistently caused the release of IL-8, a chemokine chosen for bioactivity monitoring (Fig. 1b). Significant increased in IL-8 concentrations were not observed with control viruses that did not express TRAM relative to mock infection (Fig. 1b). The increase with TRAM expressing viruses was much larger than that observed with vectors expressing another inflammatory activator, TAK1^11^. That the pro-inflammatory response was not observed in cells infected with control viruses, in which the only difference is the absence of the TRAM gene, or with a vector containing a different transgene (TAK1), provides evidence of the transgene-specific modification of the signalling response in the cells. A GFP was used in this system to confirm that the insertion of the TRAM gene in the adenovirus vector did not modify the amount of antigen expressed, an important control to confirm that the pro-inflammatory effect was due to the transgene itself rather than to an indirect effect. Antigen expression, assessed using GFP fluorescence, was similar in three infected cell lines: HeLa (Fig. 1b), NIH-3T3 and RAW264.7 (Fig. 2) irrespective of the presence of TRAM in the virus. We have however not tagged the TRAM molecule in the transgene design out of concern that any epitope tag might alter the intra-cellular activity of TRAM, thus preventing the possibility to measure directly the increase of TRAM molecules within the cells with this method.

### Co-expression of TRAM with antigen in AdHu5 bicistronic vector increases CD8+ and decreases antibody responses to the transgene in mice

To assess whether the co-expression of TRAM was associated with a modification of AdHu5-TIP EGFP-induced immunogenicity *in vivo*, groups of BALB/c mice were immunized with the mTRAM encoding virus and compared the immune responses with the non-adjuvanted counterpart under different conditions. Insertion of the murine *Tram* gene increased the CD8+ T cell responses to the Pb9 epitope (Fig. 2a, two way ANOVA, p < 0.0001 for the TRAM effect), while it did only marginally increased CD4+ T cell responses (Fig. 2b, p = 0.0272 for the TRAM effect, observed at one dose only). This effect was not restricted to the intramuscular route: The increase of CD8+ T cell response was detected both i.m. and i.d. (Fig. 2c, two way ANOVA, p = 0.008 for the TRAM effect), and its magnitude was not route-dependant (p = 0.192 for the route effect). The CD4+ T cells responses did not differ significantly by TRAM or by the route of AdHu5 injection at that dose (Fig. 2d. p = 0.223 and p = 0.45 respectively). TRAM expression also markedly reduced the antibody response to the transgene, as measured by the reduction of GFP-specific antibody titres induced by the TRAM-expressing virus with both immunization routes (Fig. 2e, two-way ANOVA, p < 0.0001 for the TRAM effect, p = 0.89 for the route effect).

### Co-expression of TRAM on the same virus as the transgene is not required to modify the transgene-specific immune responses

**Figure 3:**
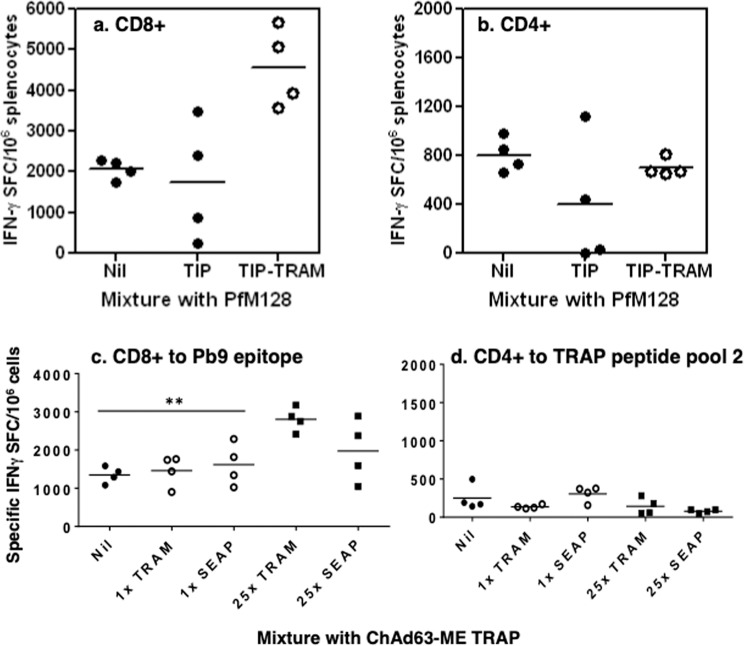
Effect of murine and human TRAM in mice by co-injection. Expression of murine TRAM impacts on the CD8+ immune response elicited by a co-injected vector. (**a,b**) Groups of C57BL/6 mice were immunized i.m. at day 0 with 10^8^vp AdHu5-Pfm128 alone (black dots, referred as Nil in the X axis) or mixed with AdHu5-TIPEGFP (black dots, referred as TIP in the X axis) or with AdHu5-TIPEGFP-tram vectors (open dots, referred as TIP-TRAM), at 10^7^ iu/mouse. Specific CD8^+^ T cell responses **(a)** and CD4^+^ responses **(b)** were measured by IFN-γ ELIspot at week 2. Each dot represents one mouse and the short lines represent the geometric means per group. Effect of human TRAM-encoding ChAd63 in mouse by co-injection **(c**,**d)**. Groups of mice were immunized i.m. at day 0 with ChAd63-ME TRAP alone or was mixed with ChAd63-hTRAM, expressing human TRAM, at a 1:1 or 1:25 ratio. **(c,d)** A control group included ChAd63-ME TRAP mixed with similar ratios of an adenovirus encoding an irrelevant antigen (secreted alkaline phosphatase, ChAd63-SEAP). Specific CD8^+^ T cell responses **(c)** and CD4^+^ responses **(d)** were measured by IFN-γ ELIspot at week 2. Each dot represents one mouse and the short lines represent the geometric means per group. Only the group that received 25x TRAM had a response significantly superior to the group that received ChAd63-ME TRAP with no adjuvant (nil).

Expression of molecules from the TLR pathway in DNA vectors can adjuvant the CD8+ T cell immune response to a transgene delivered by another, co-administered plasmid^11^, the so-called ‘*trans*’ setting. To investigate whether this effect is observed with adenovirus-based vaccines, groups of C57BL/6 mice were immunized with AdHu5-Pfm128, which encodes a malaria blood stage antigen, mixed with either AdHu5-TIP-EGFP or AdHu5-TIP-EGFP-mTRAM. C57BL/6 mice were selected for this assessment because in this strain of mice CD4^+^ and CD8^+^ T cell epitopes are present in Pfm128 that are not contained in the TIP-EGFP antigen^19^, so Pfm128 immunogenicity could be monitored without immune competition with the TIP-EGFP antigen. Two weeks after immunization, an ELISPOT assay with peptides corresponding to known Pfm128 CD8^+^ and CD4^+^ T cell epitopes was performed. The mTRAM-encoding virus significantly increased the CD8+ (Fig. 3a, p = 0.0073), but not the CD4+, T cell response to Pfm128 (Fig. 3b), a pattern of responses seen in previous experiments (Fig. 2).

### Adjuvant effect of human TRAM-encoding adenovirus in ChAd63 vector in mouse

To confirm these findings, a monocistronic chimpanzee adenovirus serotypes serotype 63 vector (ChAd63) was generated, in which the complete human TRAM open reading frame was expressed by a CMV promoter (ChAd63-TRAM). No antigen was encoded in this ‘adjuvant’ virus. It was mixed with a ChAd63 vector encoding the liver-stage malaria antigen ME-TRAP (ChAd63-ME TRAP), and antigen chosen because, when delivered by ChAd63 as part of prime boost regimen, ME-TRAM induces modest protection against malaria in field trials^20^. It also includes the pb9 epitope included in the TIPEGFP constructs.

ChAd63-TRAM was mixed with ChAd63-METRAP at 1:1 (antigen:adjuvant) or 1:25 (antigen:adjuvant) ratio, and injected into groups of mice. A control group included ChAd63-ME-TRAP mixed with similar ratios of an adenovirus encoding an irrelevant molecule (secreted alkaline phosphatase, ChAd63-SEAP), instead of the TRAM adjuvant. IFN-γ responses to the murine epitope Pb9, contained in ME-TRAP, showed that at a 1:1 ratio, human TRAM-encoding adenovirus did not increase the Pb9-specific response in mouse, while an increase was observed when ChAd63-hTRAM was in excess (1:25, Fig. 3c, p < 0.01 as compared with the group receiving no adjuvant (nil)). Mixing with ChAd63-SEAP did not significantly alter the Pb9 response induced by co-administered ChAd63-ME TRAP at any of the ratios studied. As expected, this mixture did not increase the CD4^+^ T cell responses (Fig. 3d).

### Adjuvant effect of human TRAM-encoding adenovirus in Rhesus macaques

The adjuvant effect of human TRAM-expressing vector was investigated in a non-human primate using the mixture approach described above. To this end, three groups of six Rhesus macaques were immunized with ChAd63-ME TRAP alone, or mixed with 5x ChAd63-hTRAM, or with 5x ChAd63-PfAMA1, encoding a blood-stage malaria antigen used as a control.

**Figure 4:**
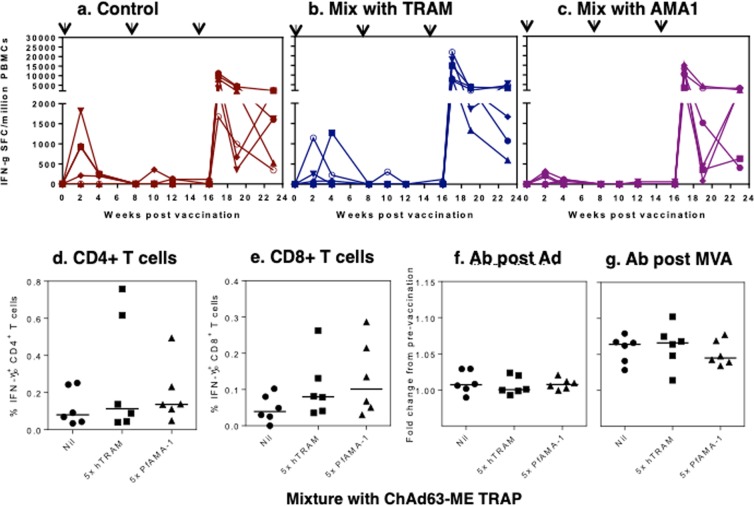
Effect of human TRAM-encoding adenovirus in Rhesus macaques. Three groups of six Rhesus macaques were immunized with ChAd63-ME TRAP alone (**a**), or mixed with 5x ChAd63-hTRAM (**b**), or with 5x ChAd63-PfAMA1 (**c**), at weeks 0 and 8. The individual TRAP-specific IFNγ responses were measured by ELISPOT at several time points as indicated. Each line represents one animal. TRAP specific IFN-γ production was measured after adenovirus vaccination (data not shown) and one week post MVA boost by intracellular cytokine staining. Graphs represent the frequency of IFN-γ^+^ CD4^+^ T cells (**d**) or IFN-γ^+^ CD8^+^ T cells (**e**) in each group with each animal displayed as a single point and lines representing the median group response. Antibodies to TRAP were measured by LIPs with the graphs representing the fold increase in TRAP responses between the pre-vaccination timepoint and 4 weeks post adenovirus prime (**f**) and 3 weeks post-MVA boost (**g**). Each animal is represented by a single point with lines representing the median response per group.

After a first injection, the TRAP-specific IFNγ ELISPOT responses peaked at week 2 or 4 and ranged from 0 to 1825 SFC/million PBMCs, with 4/6 animals responding in the control and the hTRAM-adjuvanted groups, *versus* 5/6 in the PfAMA1 group (Fig. 4a–c). No significant difference of the TRAP-specific response was observed, and in all animals, responses had declined to below detection level at week 8 (Fig. 4a–c). A second ChAd63 injection failed to boost the primary responses, as the number of IFNγ-producing cells decreased to below 400 SFU/million PBMC in responding animals (2/6 in the control and hTRAM groups, 1/6 in the PfAMA1 group, Fig. 4a–c). All animals were boosted at week 16 with MVA-ME TRAP. Strong TRAP-specific IFNγ responses were elicited in all animals one week post MVA injection, ranging from 1675 to 21890 SFC/million PBMCs. No significant difference was observed between the groups (Fig. 4a–c).

To determine the phenotype of the TRAP specific T cell response, PBMCs were restimulated overnight with TRAP peptides and IFN-γ production measured by intracellular cytokine staining. No significant difference between groups was observed for the low IFN-γ^+^ CD4^+^ or IFN-γ^+^ CD8^+^ T cells responses measured two weeks after adenovirus administration (data not shown). After the MVA boost, slightly higher frequencies of TRAP specific CD4^+^ T cells (Fig. 4d) were observed compared to CD8^+^ T cells (Fig. 4e), however there was again no significant difference in the frequency of IFN-γ^+^ CD4^+^ or CD8^+^ T cells between the groups. In addition, there was no significant difference in either the frequency or proportion of T effector, T effector memory or T central memory cells between the three groups (data not shown). TRAP antibodies were measured pre-vaccination, at week 4 post first adenovirus injection and 3 weeks post-MVA boost. While increases in TRAP specific responses were observed after vaccination, mainly after MVA boost (indicated by a greater than 1 fold increase Fig. 4f,g), no significant difference between the three groups of macaques at these two time points was observed. In conclusion, strong TRAP specific immune responses were observed in all vaccinated animals with the peak response observed one week post-MVA boost. Vaccination induced TRAP specific IFN-γ^+^ CD4^+^ and IFN-γ^+^ CD8^+^ T cells and TRAP specific antibodies, yet neither enhancement of the CD8+ response nor decrease of the antibody response by co-vaccination with ChAd63 expressing TRAM was observed.

## Discussion

In this study, the adjuvant effect of a signalling pathway molecule on a co-administered antigen previously observed with DNA immunization was investigated in the context of viral vector immunization. A modest (1.5 to 2 fold) increase of CD8^+^ T cell responses was observed in mice immunized with TRAM expressing adenovirus vaccines, concomitant with a marked decrease of antibody responses. In mice, the effect was observed in experiments where the murine *tram* gene was inserted in the same vector as the antigen, or when the antigen and TRAM (murine or human) were encoded by different vectors mixed together prior to injection. The effect was observed with the human serotype 5 as well as with the chimpanzee serotype 63 adenoviruses described above. In rhesus macaques however, there was no evidence of any adjuvant effect induced by a vector encoding human TRAM on the immune response elicited by a co-administered adenovirus, under the conditions studied. Difficulties translating murine adjuvants into primates was observed previously with 4-1BBL^21^.

The adjuvant effect was restricted to increase of CD8+ responses, while a decrease of antibody responses was observed in mouse. Such effect was previously observed with a Venezuelan equine encephalitis virus adenovirus vaccine: when IFN-alpha was co-expressed with the antigen, a reduction of transgene-specific antibody responses was observed^22^. Thus, *in vivo* interferon induction induced by TRAM^7–9^ may explain some of the effects observed.

The immune modulation induced by TRAM seen in mice was not observed in primates. The regime tested in primates used an adjuvant virus administered in a molar excess relative to the antigen expressing virus. The ratio of adjuvant to antigen expressing virus was lower than that at which significantly enhanced immunogenicity was observed in mice, but was chosen because it was considered to be a more realistic candidate for manufacturing and human use, compared with regimes with higher ratios of adjuvant to antigen (such as that used in Fig. 3a). It is possible that the mixture used was not optimal in primates.

Induction of immune responses is dependent on complex pathways, and therefore immune modulation by modification of intracellular signalling pathway may differ between species. In particular, a splice variant of TRAM, TAG, acts as a negative regulator of the TLR4 signalling pathway in primates^23^. This molecule competes with TRAM for the recruitment of TRIF, and is not found in mice, and such regulation could explain the difference of TRAM-adjuvant effect between mice and non-human primates. Therefore, it is unlikely that any modification aiming at increasing the expression of TRAM from an adenovirus vector would result in a suitable adjuvant system in human, because of the negative regulation system. However, a combination with another stimulation may overcome the negative regulation: TRAM is a membrane-anchored molecule, containing a TIR-domain adaptor that induces the MyD88-dependent signaling pathway, and ultimately activates NF-kB. We hypothesized that over-expression of TRAM could mediate intracellular signal activation, that would result in enhanced immune responses. The intracellular signal activation may be enhanced in the presence of TLR stimulation. Therefore, combining a TRAM expressing vector with TLR stimulating small molecule may enhance the adjuvant properties and vaccination efficacy. This is an area for further research.

Altogether, these results support the concept that modification of viral vector immunogenicity by modifying virally induced innate immune signalling profiles, but emphasise the difficulty of doing so in a cross-species manner. Adjuvanting viral vector induced responses in primates can be achieved by altering antigenic structure: oligomerization of several antigens with molecules such as C4bp variants^19,24^. In addition, fusion of antigens to MHC class II invariant chain also induced increased T-cell responses in mice and in non-human primates^25^. Such structural modifications of antigens may have more consistent effects between species than modulation of signalling pathways.

## Supplementary information

Supplementary Information.
